# A53 MOLECULAR MECHANISMS FOR A ROLE OF SHP-2 IN TLR4 SIGNALING IN INTESTINAL EPITHELIAL CELLS

**DOI:** 10.1093/jcag/gwad061.053

**Published:** 2024-02-14

**Authors:** V Chabot, D Lévesque, F Boisvert, N Rivard

**Affiliations:** Immunologie et biologie cellulaire, Universite de Sherbrooke, Sherbrooke, QC, Canada; Immunologie et biologie cellulaire, Universite de Sherbrooke, Sherbrooke, QC, Canada; Immunologie et biologie cellulaire, Universite de Sherbrooke, Sherbrooke, QC, Canada; Immunologie et biologie cellulaire, Universite de Sherbrooke, Sherbrooke, QC, Canada

## Abstract

**Background:**

Shp-2 gene polymorphisms are associated with greater susceptibility to ulcerative colitis. How Shp-2 contributes to disease susceptibility is however unclear. Intestinal epithelial cell (IEC)-specific deletion of *Shp-2* results in severe colitis in mice (*Shp-2*^IEC-KO^). We found important alterations in microbiota composition, namely increased *Enterobacteria* of the *Proteobacteria* phylum and decreased *Firmicutes* levels, that preceded clinical signs of inflammation. Antibiotherapy or epithelial KO of *Myd88* inhibits colitis in *Shp-2*^IEC-KO^ mice. Notably, feces from *Shp-2*^IEC-iKO^ mice display much more LPS-like bioactivity than do feces from controls (unpublished data). These observations support a role for TLR4/Myd88-bacterial recognition in initiation of colitis in *Shp-2*^IEC-KO^ mice.

**Aims:**

The aim of the present study was to identify the molecular mechanisms by which Shp-2 may regulate TLR4 signaling in IEC.

**Methods:**

1) The regulation of Shp-2 phosphorylation was analysed in IEC-6 cells stimulated or not with 10 ug/ml of LPS, a TLR4 ligand. **2)** We mapped the Shp-2-dependent interactome in LPS-stimulated IEC-6 cells by an APEX2 proximity assay used in combination with mass spectrometry (MS) analysis. Cells stably expressing APEX2-tagged Shp-2 were generated. Addition of doxycycline results in expression of “physiological” levels of Shp-2;APEX2 construct. Proximity biotinylation labeling of Shp-2 by APEX2 was performed after 10 min stimulation with LPS and labeled candidates were analyzed by MS.

**Results:**

1) Upon stimulation of IEC-6 cells with LPS, Shp-2 protein is rapidly phosphorylated (Y542, Y580) and potentially activated. This increased phosphorylation is abolished by pretreatment with N-acetyl cystein, a commonly used antioxydant. **2)** Using Prostar R package, the three replicates of MS data were filtered for the minimum quantification of 66,66% of each protein in at least one condition. They were normalized within conditions using VSN normalization, then the Partially Observed Values (POV) and the Missing in Entire Column (MEC) values were imputed respectively with SLSA and DetQuantile (Quantile 1%, Factor 0,2). A differential analysis between the LPS and non-treated conditions identified 334 proteins enriched with a FC of ± ≥2,5 with a *p*-value inferior then 0,05. Among the top Shp-2 interactors, we found Tak1 (ampersand:003E3,5X change) and Tab1 (ampersand:003E3,0X change), two proteins involved in NF-kB signaling. Decreased interaction with the TLR effectors Myd88 (ampersand:003C2,5X change) and Irak1 (ampersand:003C3,2X change) was also noted after LPS treatment.

**Conclusions:**

These data suggest for the first time that Shp-2 may regulate NF-kB signaling downstream of TLR4 in IEC. Thus, SHP-2 may contribute to the maintenance of mucosal tolerance to microbiota in the colon.

**Funding Agencies:**

CIHR

